# *Bacteroidia* and *Clostridia* are equipped to degrade a cascade of polysaccharides along the hindgut of the herbivorous fish *Kyphosus sydneyanus*

**DOI:** 10.1093/ismeco/ycae102

**Published:** 2024-08-01

**Authors:** Cesar T Facimoto, Kendall D Clements, W Lindsey White, Kim M Handley

**Affiliations:** School of Biological Sciences, The University of Auckland, Auckland, 1010, New Zealand; School of Biological Sciences, The University of Auckland, Auckland, 1010, New Zealand; Department of Environmental Science, Auckland University of Technology, Auckland, 1010, New Zealand; School of Biological Sciences, The University of Auckland, Auckland, 1010, New Zealand

**Keywords:** fish gut microbiome, cazyme, herbivory, macroalgae, metagenome, Alistipes

## Abstract

The gut microbiota of the marine herbivorous fish *Kyphosus sydneyanus* are thought to play an important role in host nutrition by supplying short-chain fatty acids (SCFAs) through fermentation of dietary red and brown macroalgae. Here, using 645 metagenome-assembled genomes (MAGs) from wild fish, we determined the capacity of different bacterial taxa to degrade seaweed carbohydrates along the gut. Most bacteria (99%) were unclassified at the species level. Gut communities and CAZyme-related transcriptional activity were dominated by *Bacteroidia* and *Clostridia*. Both classes possess genes CAZymes acting on internal polysaccharide bonds, suggesting their role initiating glycan depolymerization, followed by rarer *Gammaproteobacteria* and *Verrucomicrobiae*. Results indicate that *Bacteroidia* utilize substrates in both brown and red algae, whereas other taxa, namely, *Clostridia*, *Bacilli*, and *Verrucomicrobiae*, utilize mainly brown algae. *Bacteroidia* had the highest CAZyme gene densities overall, and *Alistipes* were especially enriched in CAZyme gene clusters (*n* = 73 versus just 62 distributed across all other taxa), pointing to an enhanced capacity for macroalgal polysaccharide utilization (e.g., alginate, laminarin, and sulfated polysaccharides). Pairwise correlations of MAG relative abundances and encoded CAZyme compositions provide evidence of potential inter-species collaborations. Co-abundant MAGs exhibited complementary degradative capacities for specific substrates, and flexibility in their capacity to source carbon (e.g., glucose- or galactose-rich glycans), possibly facilitating coexistence via niche partitioning. Results indicate the potential for collaborative microbial carbohydrate metabolism in the *K. sydneyanus* gut, that a greater variety of taxa contribute to the breakdown of brown versus red dietary algae, and that *Bacteroidia* encompass specialized macroalgae degraders.

## Introduction

Some marine herbivorous fishes rely on symbiotic relationships with microbial gut communities for nutrition and health [1, 2]. Significant levels of fermentation products are found within the gut of herbivorous taxa such as kyphosid chubs and unicornfishes of the genus *Naso*, and turnover rates of the resulting short-chain fatty acids (SCFAs) are comparable to those in herbivorous mammals and reptiles [1]. These factors emphasize the importance of microbial fermentation in supporting the energetic metabolism of host fishes. However, hindgut fermentation appears to be metabolically important in only a few herbivorous fish species, all of them marine [3, 4]. In particular, the family *Kyphosidae* exhibits the highest levels of SCFA measured to date [3, 5]. Of the *Kyphosidae*, *Kyphosus sydneyanus* (Silver Drummer) displays exceptional levels of SCFA that are among the highest levels of SCFAs measured in the gut of any fish to date [1, 4–6], highlighting a significant role for microbial gut communities in this species. Studies on endogenous activities (foregut wall tissue enzymatic extracts) of herbivorous fish conducted to date indicate that *K. sydneyanus* can only produce amylases [2, 7, 8] and perhaps laminarinases [2, 7]. Therefore, complex polysaccharides appear largely resistant to foregut endogenous enzymes and likely fuel hindgut microbial fermentation.

Investigation of the stomach contents of *K. sydneyanus* has revealed a prevalence of the brown algae species *Carpophylum maschalocarpum* (constituting 10%–50% of stomach content) and *Ecklonia radiata* (15%–50% of stomach content) [2]. Additionally, *Gigartina macrocarpa* (5%–30% of stomach content) and *Caulacanthus ustulatus* (up to 15% of gut content) represent common red algae species present [2]. Therefore, the gastrointestinal tract of *K. sydneyanus* is enriched with a diverse array of carbohydrates such as alginate, laminarin, mannitol, and fucose-containing sulfated polysaccharides (FCSP) present in brown algae species [9]. In addition, the gut contains galactans such as carrageenan and agarose and α-glucans such as floridean starch from dietary red algae [8, 10–12]. These substrates likely constitute the bulk of fermentation substrates supplied to gut microbial communities and govern metabolism along the fish gut.

Recent work on the composition of the hindgut microbiota in *K. sydneyanus* revealed the predominance of the phyla *Bacteroidota* and *Bacillota* across 60 individuals [13]. The relative abundances of these phyla differ longitudinally along the fish gut from mid to distal sections [13, 14], and certain families within the *Bacillota*, such as *Lachnospiraceae*, *Erysipelatoclostridiaceae*, *Oscillospiraceae*, and *Acholeplasmataceae*, are significantly more abundant in the midgut [6, 13]. In contrast, the phylum *Bacteroidota* exhibits a gradual increase in relative abundance from the mid to distal gut and is primarily represented by the family *Rikenellaceae* [6, 13, 14]. These observed variations in community composition across different gut sections, along with differing levels of SCFA, indicate regional variation in gut metabolism [6].

Despite longitudinal shifts in microbial composition, metagenomic investigation of the *K. sydneyanus* hindgut using unbinned contigs indicated the conservation of metabolic pathways along the gut [14], including redundancy in the metabolic capacity for algal degradation. Further metagenomic analysis of other *Kyphosus* species (e.g., *K. vaigiensis*, *K. hawaiiensis*, and *K. cinerascens*), also using unbinned contigs, revealed the enrichment of agarases, porphyranases, carrageenases, ulvanases, and alginate-lyases primarily supplied by *Bacteroidota* [15]. Consistent with these findings, enrichment cultures from *K. vaigiensis, K. hawaiiensis* have recently been shown to encode agarases, carrageenases, and porphyranases involved in the degradation of red algae and alginate lyases and fucoidanases involved in the degradation of brown algae [16]. Moreover, isolation of some of the abundant members of the *K. sydneyanus* gut community (*Bacillota*, *Tannockella kyphosi*, and *Chakrabartyella piscis*) [17, 18] demonstrated the capacity of these bacteria to utilize mannitol—a sugar alcohol that is the major storage metabolite of brown algae, is fermented in the *K. sydneyanus* gut, and is not metabolized by vertebrates [19, 20]. A detailed understanding of how such metabolic capacities for algal substrate degradation are distributed among the hindgut microbiota of *Kyphosus* species, including *K. sydneyanus*, is yet to be determined.

Here, we investigated the distribution of carbohydrate degradation capacity among the *K. sydneyanus* hindgut microbiota. We focus our study on *K. sydneyanus* due to the exceptionally high levels of SCFAs measured along the gut of this fish species [1]. Additionally, this species is unique among hindgut-fermenting fish species for the amount of contextual background data available, enabling the metabolic potential of the gut community to be interpreted alongside: (i) information on *ex vivo* rates of fermentation in this species [1] (lacking for the vast majority of fish species) and the metabolic capacity of the host to utilize fermentation products [21]; (ii) detailed data on the dietary algae of the species (including ontogenetic and seasonal variation in dietary algae) [2], allowing us to predict the availability of carbohydrate substrates; and (iii) detailed data on microbiota composition for this species, including variation among individuals [6, 13, 14].

To determine the capacity of different hindgut taxa to degrade dietary macroalgal substrates along the fish gut, we generated metagenome-assembled genomes (MAGs) and metatranscriptomes from different longitudinal sections of the hindgut lumen. Next, we determined the carbohydrate active enzyme (CAZyme) repertoire encoded by these genomes and the capacity to breakdown polysaccharides, particularly those comprising the *K. sydneyanus* diet of brown (e.g., *C. maschalocarpum* and *E. radiata*) and red (e.g., *G. macrocarpa*) algae [2]. This included the polysaccharides alginate, laminarin, and FCSP that are important constituents of brown algae [10, 11, 22, 23], as well as carrageenan, the major cell wall polysaccharide in *Gigartina* species [12]. Our findings shed light on the differentiation and redundancy of carbohydrate utilization mechanisms among the gut microbiota, and highlight the potential for cooperative dynamics, including the initiation of glycan degradation by *Bacteroidia* and *Clostridia*, and variation in taxon preferences for brown and red algal substrates.

## Materials and methods

### Sample collection and metagenomic and metatranscriptomic sequencing

Four *K. sydneyanus* individuals were collected in the summer of 2017 by underwater spear on snorkel from waters adjacent to the neighbouring Great Barrier (Aotea) and Little Barrier (Hauturu) islands in north-eastern New Zealand (Table S1). An additional six *K. sydneyanus* individuals were collected via the same method in summer 2020 from Great Barrier Island. The initial four fish were used for generating metagenomes, and all analyses relate to these four fish unless otherwise specified. The six fish from 2020 were used for metagenomics and metatranscriptomics, and metagenomic results between the 2017 and 2020 are compared to illustrate the high reproducibility of results across individual fish and sampling years (Figs S1 and S2). Fish collections from 2017 and 2020 were covered by approvals 001636 and 001949 from the University of Auckland Animal Ethics Committee. Gut sampling for metagenomic and metatranscriptomic sequencing was conducted as described previously [14] and is summarized below. First, the gastrointestinal tract of each fish was separated immediately after capture and divided into sections [1]. The stomach was assigned as section I and the hindgut chamber as section V. The remaining intestine was divided into three sections of equal length and designated as II (immediately after the stomach), III, and IV (immediately before the hindgut sphincter). Lumen contents from each section were homogenized and preserved by flash freezing with liquid nitrogen immediately upon division of gut sections.

DNA from the gut lumen contents was extracted from all 10 fish, including gut sections IV and V (from fish collected in 2017 and 2020) and section III (from fish collected in 2020). DNA from fish collected in 2017 was obtained using the PowerSoil DNA extraction kit (Mo Bio Industries, Ltd., Carlsbad, USA), and DNA from fish collected in 2020 was obtained using the PowerSoil Pro kit (QIAGEN, Germantown, MD, United States). DNA quality was check by gel electrophoresis. All 8 samples from 2017 (two gut sections per fish) [14] and 18 from 2020 (three gut sections per fish) were used for metagenomics. RNA was extracted from the lumen contents of the six 2020 fish using the Monarch Total RNA Miniprep Kit (New England BioLabs, Ipswich, MA, United States) including a DNA removal step. Samples for metatranscriptomics were selected based on detectable RNA of good quality (RIN values >5) determined with the Agilent Bioanalyzer and RNA 6000 Nano kit (Agilent Technologies, Santa Clara, CA, USA), and comprised: three section III samples (i.e., from three fish), four section IV samples (i.e., from four fish), and all section V samples (i.e., from six fish).

Metagenome and metatranscriptome generation, MAG reconstruction, and transcript mapping to MAGs are described in Supplementary Information.

### Taxonomy and phylogenetic tree

Taxonomic classification of dereplicated MAGs (sharing <99% average nucleotide identity, see Supplementary Information and Table S2) was undertaken using the Genome Taxonomic Database Toolkit (GTDB-Tk) version 2.1.1 with the 214 database release [24]. A phylogenetic tree was produced with the protein sequence alignment file provided by GTDB-Tk and with IQ-TREE version 1.6.12 (parameters -m TEST -b 1000) [25] and reference genomes from GTDB (Tables S3 and S4).

### Gene prediction and annotation

Genes were predicted with PRODIGAL version 2.6.3 using meta mode [26]. Protein sequences were annotated using USEARCH version 9.02132 [27] against UniRef100 release 2018_09 [28], UniProt (SwissProt and TrEMBL) release 2018_09 [29], and KEGG release 86.1 [30] databases, considering best hits with at least 50% with reference coverage by length, 30% identity, and an e-value of 0.001. A protein domain search using HMMER v3.1b2 [31] was performed against Pfam version 32.0 [32] and TIGRFAM version 14.0 [33] (0.001 e-value cutoff).

CAZymes were annotated using dbCAN version 3.0.7 [34] using the protein mode and outputting CAZyme gene clusters (CGCs). A CAZyme annotation was considered correct if at least two of the dbCAN output annotations (e.g., HMMER, eCAMI, or DIAMOND) matched. The final CAZyme annotation prioritized HMMER annotations followed by eCAMI. The inference of CGCs substrate specificities were based on the presence of dedicated CAZy families and/or their colocalization with CAZy families containing complementary activities (Supplementary Information).

A BLAST-like DIAMOND search was also performed against the SulfAtlas version 2.3.1 database [35] and a subset of proteins from CAZy database version 3.0.7 [34] containing only proteins assigned to an EC number. The search was performed with DIAMOND version 2.0.15 [36] using blastp mode with a e-value cut off of 0.001. Sulfatases were only considered if the database target coverage was ≥50%, sequence identity ≥30%, and annotations contained the protein domain ‘PF00884’ [37]. The annotation of ECs associated with the degradation of brown and red algal polysaccharides is detailed in Supplementary Information, and EC numbers and substrate predictions are shown in Table S5. CAZymes associated with an EC number were only considered if the database target coverage was ≥40% and identity ≥30%. Details about the curation of mannitol-associated genes are in Supplementary Information and Table S6. CAZyme, sulfatase and mannitol annotations are given in Table S7, and CGC annotations are shown in Tables S8–S11.

### Statistical analysis and average nucleotide identity calculations

Statistical analyses were carried out with R version 4.1.1 [38]. A Wilcoxon rank-sum test was performed to determine CAZyme density across bacterial classes using the ggpubr package [39]. Bray–Curtis dissimilarities were constructed using the vegan package [40] with MAG CAZyme densities. The package rstatix [41] was used to calculate statistical differences in relative abundance of genera across sections, and average predicted number of CGCs per genus, using the Kruskal–Wallis test. To identify clusters of co-abundant MAGs, pairwise Pearson correlation coefficients of MAG relative abundances were calculated using the rcorr function from the Hmisc package [42] and the heatmap was generated using corrplot [43]. Average Nucleotide Identities (ANI) between *Alistipes putredinis*, *Alistipes communis*, and *Alistipes* MAGs from this study were calculated using the ANI calculator (http://enve-omics.ce.gatech.edu/ani/) [44].

## Results and discussion

### Widely distributed carbohydrate degradation capacity among phyla in the *K. sydneyanus* hindgut

**Figure 1:**
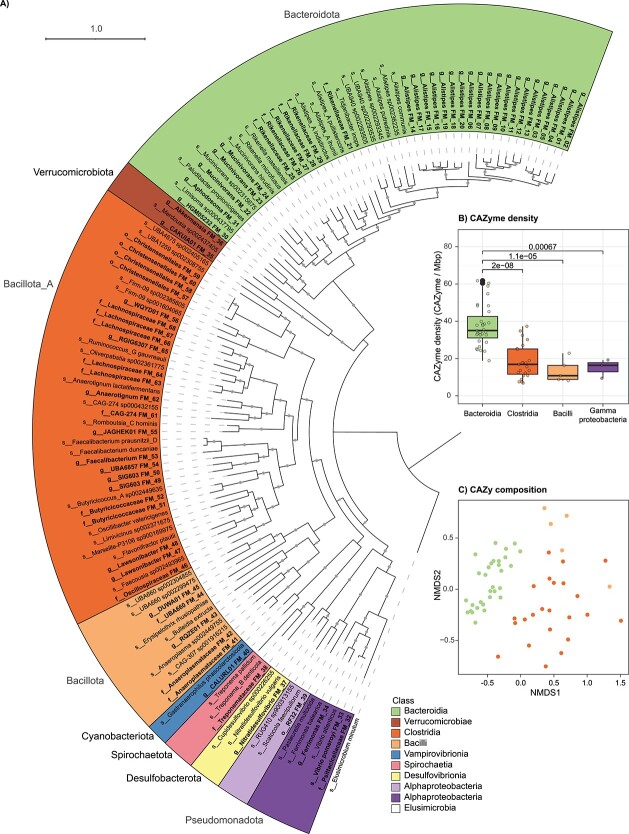
Phylogeny and CAZyme composition of the 68 representative MAGs. (A–C) Taxa clades and CAZyme density and composition are coloured by taxa class (see key). (A) Phylogenetic tree of MAGs and references based on the alignment of 120 conserved bacterial markers. Genomes are labelled with their lowest taxonomic resolution classified. Refined MAGs are indicated by an MAG ID and are displayed in bold, and reference genomes are shown unbolded. Phyla classifications determined by GTDB-Tk are indicated by outer labels. Bootstrap values above 70 are shown as solid gray circles. Tree scale is shown at the top of the plot. (B) Boxplots displaying the CAZyme density of *Bacteroidia*, *Clostridia*, *Bacilli*, and *Gammaproteobacteria* classes. Upper brackets indicate the significance (*P* values <0.05). The boxes and central line represent the interquartile range and median CAZyme density across MAG in the class, and the whiskers represent the minimum and maximum values within 1.5 times the interquartile range. (C) Non-metric multidimensional scaling (NMDS) of CAZyme composition in *Bacteroidia*, *Clostridia*, and *Bacilli* MAGs.

A total of 197 MAGs were recovered from the eight gut content samples (sections IV and V) from the initial set of four fish. Of these, 68 representative MAGs (75.0%–99.5% complete and 0%–6.4% contaminated) were retained for further analysis after cross-sample dereplication (99% ANI threshold) and refinement (Tables S2 and S3), meeting MIMAG medium–high- and high-quality criteria based on completeness and contamination [45]. The percentage of total metagenomic reads mapped to these MAGs ranged from 7.4% to 39.4% (see Table S1 for prokaryote versus eukaryote read distribution estimates). Following taxonomic classification most representative MAGs (*n* = 63) were assigned to a family, whereas only 43 were further classified to a genus. Approximately 37% of MAGs from the fish gut microbiome therefore belong to previously unidentified genera. MAGs were distributed across eight phyla (Fig. 1A). *Bacteroidota* encompassed the highest number of MAGs (*n* = 31 all class *Bacteroidia*), followed by *Bacillota*_A (*n* = 23, all class *Clostridia*) and *Bacillota* (*n* = 5, all class *Bacilli*). The phylum *Bacillota* is split into multiple groups based on genome phylogeny in GTDB (e.g., *Bacillota* and *Bacillota*_A), and represent distinct clades based on our analyses (Fig. 1A). The remaining nine representative MAGs, or populations, belonged to five other phyla (*n* = 4 *Pseudomonadota*, *n* = 2 *Verrucomicrobiota*, and one each of *Cyanobacteriota*, *Spirochaetota*, and *Desulfobacterota*).

**Figure 2:**
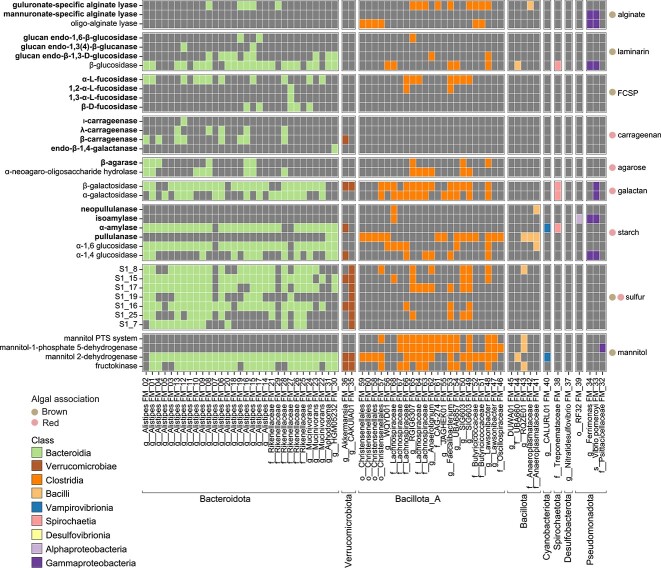
Heatmap showing the presence of genes associated with carbohydrate degradation in each MAG. Gene presence is indicated by boxes coloured by taxonomic assignment at the class level, and absence is indicated by grey shading. MAGs, shown along the *x*-axis, are ordered by their phylogenetic placement in Fig. 1A. Labels at the bottom indicate phyla. Predicted enzymes are denoted on the *y*-axis on the left, and predicted substrates are shown on the right (Table S5). Predicted CAZymes known to act on HMW polymers (based on their EC number) are shown in bold. The upper seven plot panels display the presence of CAZymes targeting the main polysaccharides of brown and red algae (panels alginate to starch). The eighth panel displays the presence of sulfatases associated with FCSP and carrageenan desulfation. The bottommost panel shows the presence of genes associated with mannitol utilization.

These eight phyla exhibited widely differing capabilities for the degradation of carbohydrates associated with dietary algae (i.e., alginate, laminarin, FCSP, carrageenan, agarose, FCSP/carrageenan/agarose derived galactan, starch, FCSP/carrageenan/agarose-bound sulfur, and mannitol). Members of each phyla encoded enzymes targeting from zero (*Desulfobacterota*) to eight of these substrate types (*Bacteroidota*) (Fig. 2). *Bacteroidota* and *Bacillota*_A (targeting all substrate types) were not only the most numerous bacteria in the fish hindgut but also collectively targeted the largest range of substrate types, highlighting the importance of these phyla in the breakdown of algal polysaccharides and metabolism of mannitol. This is further reflected by the high CAZyme gene densities of members of these two phyla (classes *Bacteroidia* and *Clostridia*, Fig. 1B). However, the genetic repertoires of five of the six remaining phyla (*Bacillota*, *Pseudomonadota*, *Verrucomicrobiota*, *Cyanobacteriota*, and *Spirochaetota*) suggest that these other phyla, when taken together, can also contribute to the breakdown of all the dietary substrates with the exception of fucoidan and agarose (Fig. 2). Of these, *Pseudomonadota* (*Ferrimonas* and *Vibrio* MAGs) are predicted to degrade four of the seven polysaccharides (alginate, laminarin, galactan, and starch) and have CAZyme gene densities broadly equivalent to *Bacillota*_A (*Clostridia* versus *Gammaproteobacteria*, Fig. 1B). *Verrucomicrobiota* (*Akkermansia* and CAKUIA01 MAGs) are likewise important for encoding a wide range of sulfatases associated with FCSP and carrageenan desulfation. Overall, most of the degradative capacity harboured by these five other phyla is associated with polysaccharides from brown algae (i.e., alginate, laminarin, and FCSP) rather than red algae (i.e., carrageenan and agarose) (Fig. 2). Accordingly, analysis of transcriptional data indicates a role for these other phyla in the degradation of brown algae, along with starch or glycogen (discussed further below).

### *Bacteroidia* and *Clostridia* are the major CAZyme-encoding classes in the *K. sydneyanus* hindgut

The comparatively large number of *Bacteroidota*, *Bacillota*_A and *Bacillota* MAGs recovered in this study (i.e., 59 out of 68 representative MAGs) is consistent with (i) our previous 16S rRNA gene analysis of 60 *K. sydneyanus* fish luminal samples [13], (ii) analysis of the unassembled version of the metagenomic dataset [14], which captured more of the rarer community (Table S1), and (iii) our expanded analysis of 397 dereplicated MAGs of 10 fish (2017 and 2020 datasets), which showed no significant differences in the relative abundances of the main taxonomic groups (e.g., *Bacteroidia*, *Clostridia*, and *Bacilli*, Fig. S1A) between the two sample collections, and highlights the importance of these taxonomic groups to *K. sydneyanus* as well as their stability through time.

The *Bacillota*_A comprised the families *Lachnospiraceae*, *Oscillospiraceae*, and *Acutalibacteraceae* (Fig. 1A, Table S3). *Bacillota*_A is recognized for its fibrolytic capacity in the mammalian gut, in which species of *Oscillospiraceae* and *Lachnospiraceae* are well-established starch and cellulose degraders [46–48]. As butyrate producers, these families are also associated with host health and nutrition [48, 49]. MAGs from *Clostridia* class (*Bacillota*_A) represented the second most (or second equal) CAZyme dense group (Figs 1B and S1B and C), indicating they harbour less genetic capacity for glycan metabolism than the *Bacteroidia* (*Bacteroidota*). This is consistent with prior studies showing comparatively fewer CAZymes encoded by members of the phylum *Bacillota* than *Bacteroidota* [50, 51]. Nonetheless, we found that both *Bacteroidia* and *Clostridia* contributed substantially more to overall community gene transcription, and CGC transcription, than any other taxa group in the hindgut chamber (section V, Fig. S2), which is where the highest microbial biomass and levels of SCFAs occur [6].

The significance of *Bacteroidota* in complex polysaccharide metabolism has been demonstrated in the gastrointestinal tract of vertebrates [52, 53], soil [54], and marine environments [37, 55–57]. In these niches, *Bacteroidota* are key players in carbohydrate biomass recycling due to their large repertoire of CAZymes, CGCs, and transporters dedicated to the saccharification of specific complex carbohydrates [55, 57, 58]. In the present study, *Bacteroidia* exhibited a significantly higher density of CAZymes compared to other taxonomic classes (Fig. 1B). This observation was consistent across the gut communities of fish collected in 2017 and 2020 (Fig. S1B and C). *Bacteroidia* also exhibited a distinct CAZyme composition (Fig. 1C), implying that members of this bacterial class have a distinct carbohydrate utilization capacity. Among *Bacteroidia* MAGs, the genus *Alistipes* (*Rikenellaceae*) represented the largest taxonomic group (*n* = 20/31 MAGs), followed by unclassified *Rikenellaceae* (*n* = 6/31 MAGs). The *Alistipes* formed a phylogenetically distinct clade from other *Bacteroidia* and *Alistipes* from other habitats (Fig. 1A). The closest cultivated relatives to this clade were *Alistipes communis* and *Alistipes putredinis*, both of which are primarily described from human clinical studies [59] (72.2%–75.7% ANI shared with 16 *Alistipes* MAGs, Table S4).

Increases in the relative abundance of *Alistipes* in the human gut have been attributed to animal protein–rich diet treatments (e.g., eggs, bacon, pork, and beef) [60], implying the involvement of this genus in protein degradation. In mice, plant-based diets (e.g., comprising resistant maltodextrin, fructo-oligosaccharide, galacto-oligosaccharides, and iso-malto-oligosaccharides) are associated with a decline in the relative abundance of *Alistipes* [61, 62]. In contrast, metagenomic studies on the rumen suggest *Alistipes* have the capacity to utilize oligosaccharides from plant-cell walls. For example, *Alistipes* increased in relative abundance in rumen metagenomes of Holstein cows fed on a high-forage (HF) diet and also displayed higher abundances of glycoside hydrolases (GHs) and carbohydrate binding modules (CBMs) [63]. The predominance of *Alistipes* within the large intestine of ruminants has also been attributed to the utilization of host glycan, based on high numbers of CAZy families GH109, GH20, and GH92 encoded by these organisms [64]. Whereas the role of *Alistipes* in many host taxa remains under-explored, studies collectively suggest that species within this genus perform differing host-dependent roles related to substrate degradation [59]. *Alistipes* in *K. sydneyanus* are thus predicted to degrade the polysaccharides of seaweeds in the host diet, and their potential to do so, alongside other carbohydrate degraders, is discussed below.

### *Bacteroidia* and *Clostridia* species are capable of initiating polysaccharide degradation

Genomic analyses revealed that the microbial community in the hindgut of *K. sydneyanus* encoded a diverse array of enzymes to attack complex carbohydrates in the fish diet (Fig. 2) and completely degrade these to monosaccharides (e.g., glucose, galactose, fucose, and uronic acids). However, enzymes acting on high-molecular-weight (HMW) substrates, such as guluronate-specific alginate lyases; glucan endo-1,6-β-glucosidases; glucan endo-1,3-β-D-glucosidases; endo-1,3(4)-β-glucanases; α-fucosidases; κ-, ι-, and λ-carrageenases; and β-agarases were found primarily within a small number of *Bacteroidia* and *Clostridia* MAGs (the exception being a guluronate-specific alginate lyase encoded by a single *Bacilli* and two *Gammaproteobacteria* MAGs encoding a mannuronate-specific alginate lyase and an oligo-alginate lyase each) (see bolded enzymes, Fig. 2). The capacity for initiating the hydrolysis of HMW polysaccharides into smaller polymers for membrane transport is a critical step in enabling the utilization of dietary substrates [65]. Altogether, the substantial representation of *Bacteroidia* and *Clostridia* within the gut communities, alongside their enzymatic capacity for cleaving internal molecule bonds (endo-acting capacity), suggests that they play a pivotal role in the degradation of substrates derived from the fish diet. This observation is in line with the current status of *Bacteroidota* as efficient polysaccharide degraders in diverse environments [51, 56, 66–68] and other *Kyphosus* species [15]. However, this result also suggests the microbial drivers of *Kyphosus* seaweed degradation are somewhat unique. Studies on other herbivorous fish species, such as the surgeonfishes *Acanthurus sohal*, *Naso elegans*, and *Naso unicornis*, attributed the cleavage of dietary polysaccharides primarily to *Bacillota*_A [69, 70] encoded endo-β-1,4-glucanases (GH74), β-agarases (GH50 and GH86), and β-porphyranases/κ-carrageenases (GH16) [69, 70].

Encoded enzymes targeting low-molecular-weight (LMW) substrates such as β-glucosidases, β-galactosidases, and α-galactosidases were widely distributed across genomes within *Bacteroidia* and *Clostridia* (Fig. 2), and across other classes (*Bacilli*, *Gammaproteobacteria*, *Spirochaetia*, and *Verrucomicrobiae*). Of these, β-glucosidase genes are associated with the utilization of oligosaccharides derived from laminarin, although both β- and α-galactosidases are implicated in the downstream degradation of galactose-rich polysaccharides such as FCSP, carrageenan, and agarose. These are the degradation products of substrates acted on by predicted HMW targeting enzymes encoded by members of the same microbial community. Hence, a larger number of MAGs containing only LMW acting capacities likely depended on the extracellular hydrolyzation of HMW substrates undertaken by a small number of keystone *Bacteroidia* and *Clostridia* in order to grow [71].

Encoded enzymes associated with the hydrolysis of starch, including HMW (e.g., neopullulanases, isoamylases, α-amylases, and pullulanases) and LMW forms (e.g., α-1,6 glucosidases and α-1,4 glucosidases), were both widely distributed in the community (Fig. 2). However, the utilization of starch is unlikely to be a major component of adult *K. sydneyanus* microbial metabolism, as starch constitutes <3% (dry weight) of the nutritional composition of dietary red algae such as *Gigartina livida* [8]. Also, the effect of acid lysis in the *K. sydneyanus* stomach, in addition to the high activity levels of amylases found in the anterior intestine (>300 μg sugar reduced ml^−1^ min^−1^ in section II), may digest starch completely or leave very little amounts of starch in later portions of the gut [7, 8].

Polysaccharides such as FCSP and carrageenan are heavily associated with sulfate esters (up to 40% of its dry weight) [72–74]. To consume these polysaccharides, the sulfate groups in the sugar backbone require removal prior to hydrolysis [73, 75]. Released sulfate can then be utilized as an energy source by sulfate-reducing bacteria or converted into sulfur-containing amino acids by the community [76, 77]. In the present study, *Bacteroidia* MAGs, along with two *Verrucomicrobiae* MAGs (primarily MAG CAKUIA01 FM_35), encoded an enriched and expanded repertoire of sulfatase families associated with FCSP and carrageenan desulfation (Fig. 2, Table S7). These functions were comparatively scarce in genomes from *Clostridia* and almost entirely lacking from other taxa (just one was encoded by a single *Bacilli* MAG). Among the sulfatase families identified, S1_15, S1_16, and S1_17 are commonly associated with sulfate removal from FCSP derived from *Fucus vesiculosus* (sulfation on positions C2 and C3) or *Cladosiphon okamuranus* (sulfation on position C4) [73]. S1_25 is an exo-sulfatase acting on position С3 of fucose [78]. *Bacteroidia* and *Verrucomicrobiae* alone possessed S1_7 and S1_19. The ι- and κ-carrageenan isoforms depend on sulfatase families S1_7 or S1_19 to remove sulfate esters on position C4 of its galactose blocks [79]. Other sulfatases required for carrageenan breakdown were more widely distributed. The ι-carrageenan requires prior removal of sulfate esters on position C2 of 3,6-anhydro-D-galactose moieties by sulfatase family S1_17 [79, 80], which was found in some *Clostridia* MAGs alongside those in *Verrucomicrobiae* and numerous *Bacteroidia* MAGs. Lastly, the sulfatase family S1_8 is implied to perform the desulfation on position C2 of the galactose moieties of λ-carrageenan [81] and was also present in some *Clostridia* MAGs and a single *Bacilli* MAG. Altogether, the enrichment of genes necessary for HMW sulfated polysaccharide depolymerization further emphasizes the key role predicted for *Bacteroidia* in initiating the deconstruction of polysaccharides from the fish diet.

The ability of the microbiota to ferment mannitol likely underpins the complete depletion of this sugar alcohol, as observed along the gut of *K. sydneyanus* [19]. This sugar alcohol could support the growth of microbes with a limited capacity to consume complex carbohydrates in more proximal sections of the fish gut [19]. However, not all bacteria with the capacity to use mannitol may do so in *K. sydneyanus*. *Chakrabartyella piscis* (*Clostridia*, *Lachnospiraceae*) isolated from the *K. sydneyanus* gut, can grow on mannitol as a carbon source (mean growth rate 1.30 ± 0.45 SD × 10^5^ CFU/h) [20], although mannitol fermentation was not detected in the presence of gut fluid [18], suggesting prioritization of other sugars in the gut fluid by *C. piscis*. In this study, we found that genes for mannitol utilization via mannitol 2-dehydrogenase (M2DH) and fructokinase were mainly present, and widespread, among *Bacteroidia* and *Clostridia* (along with two *Verrucomicrobiae* MAGs, Fig. 2) enabling conversion of D-mannitol to phosphorylated fructose (D-fructose-6P) in the glycolysis pathway. Of these taxa, only the *Clostridia* (alongside a *Bacilli* and a *Gammaproteobacteria* MAG) encoded mannitol phosphotransferase system (PTS) components and/or mannitol-1-phosphate 5-dehydrogenase (M1PDH) genes (Fig. 2) [82, 83], which together facilitate another route from D-mannitol to D-fructose-6P, independent of *Bacteroidia*. This result is consistent with *Clostridia* found in surgeonfishes of the genera *Acanthurus* and *Naso*. In these fishes, abundant intestinal *Lachnospiraceae* are capable of uptake mannitol via the PTS system [69, 70]. Some *Clostridia* MAGs in this study (and the *Bacillota* MAG) encoded genes involved with both M2DH and PTS- M1PDH pathways for mannitol utilization, including unclassified *Christensenellales*, *Lachnospiraceae*, and *Anaerotignum*, UBA6857, *Faecalibacterium*, SIG603 and *Lawsonibacter* genera (Fig. 2). The presence of the PTS-M1PDH system or both pathways for mannitol utilization in some taxa that are dominant in gut sections preceding the hindgut chamber may confer a competitive advantage to *Bacilli* in section III and *Clostridia* in section IV for mannitol usage.

### *Alistipes* (*Bacteroidia*) are CAZyme gene cluster–rich and predicted to be specialized degraders of polysaccharides

**Figure 3:**
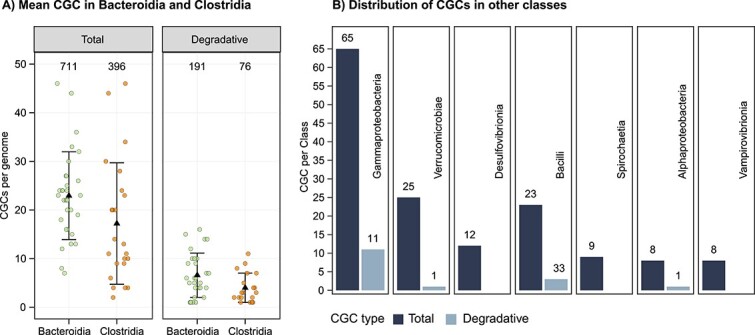
Summary of CGC types across class and their degradative CAZyme composition. (A) Strip plots showing the total and degradative number of CGCs encoded per MAG in *Bacteroidia* (green) and *Clostridia* (orange). Degradative CGCs are defined here as those containing at least two degradative CAZymes. Black triangles indicate the mean of CGCs per class (across MAGs) and error bars show the standard deviation. Numbers at the top display the sum of each CGC type per taxonomic class. (B) Bar plots indicating the sum of total and degradative CGCs in other classes, which each have <3 MAGs containing degradative CGCs. Numbers above bars show the CGC count.

**Figure 4:**
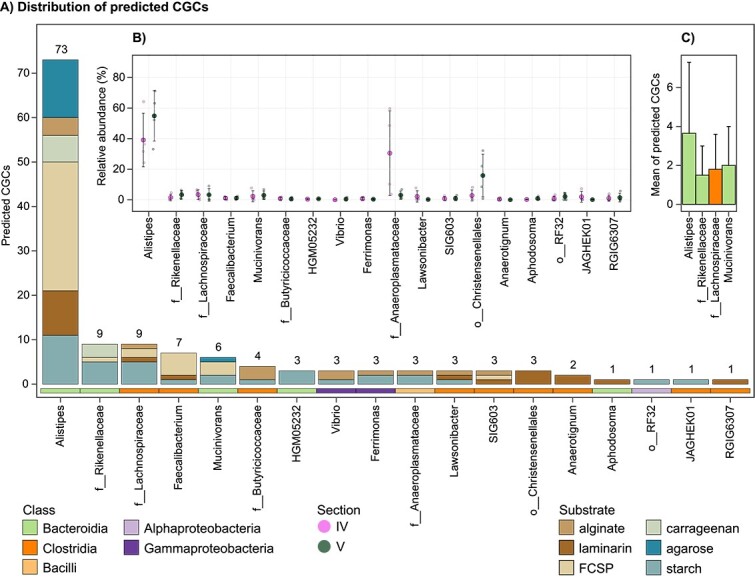
Classified CGCs with degradative properties associated with substrates common to the fish diet. (A) Number of predicted CGCs per genus. Stacked bars are coloured according to their predicted substrate. Tiles below bars indicate taxa class affiliations. (B) Relative abundances in sections IV and V of genera containing predicted CGCs. Small open circles represent the genus relative abundance in each fish. Mean relative abundance values are shown by large circles coloured by sections, and error bars denote standard deviations. (C) Bar plot of the average number of predicted CGCs per MAG in the top four genera with more than two MAGs. Bars are coloured by class affiliation and error bars indicate the standard deviation.

Investigation of carbohydrate-acting gene arrangements can provide valuable information for elucidating the degradation pathways for specific glycans and discovering CAZyme families [84]. CAZymes encoded by the same CGC generally feature a set of complementary enzymes capable of fully deconstructing a specific glycan (e.g., a glucan endo-1,6-β-glucosidase and β-glucosidase to hydrolyse laminarin into glucose) (Table S8) and as such, facilitate glycan degradation via their co-expression [34, 57, 71]. Analysis of the *K. sydneyanus* gut community revealed a total of 1257 CGCs, in which 283 contained at least two degradative CAZymes (e.g., GH, and/or PL, and/or CE) (Fig. 3A and B). Among these degradative CGCs, 135 could be linked to substrates relevant to the fish diet including alginate, laminarin, FCSP, carrageenan, agarose, and starch (Fig. 4A). Accordingly, these CGCs often encoded enzymes such as alginate lyases (PL17, PL34, or PL6), laminarinases (GH30, GH149, or GH16), fucosidases (GH29, GH30, or GH141), carrageenases (GH150), agarases (GH16 or GH86), and amylases (GH13, GH133, GH57, or GH77) (Table S9).

Inspection of substrate-assigned CGC distributions across the gut communities (associated with the *K. sydneyanus* diet) revealed a sizeable and abundant collection of CGCs within the genus *Alistipes* (*n* = 73, across 20 MAGs) (Fig. 4A). Per MAG, the average number of CGCs in *Alistipes* was 3.65 ± 3.34 SD (followed by *Mucinivorans* with 2.00 ± 1.00 SD algae-associated CGCs per MAG) (Fig. 4C, Table S10). Results imply both redundancy and diversity in the carbohydrate utilization pathways employed by *Alistipes*, considering the distribution of genes with CAZyme-encoding functions across the group (guluronate-specific alginate lyases = 6, glucan endo-1,3-β-D-glucosidases = 13, α-L-fucosidases = 79, κ-carrageenases = 8, and λ-carrageenases = 5) (Table S11). In addition to CGC diversity in *Alistipes*, the genus displayed considerable variation in relative abundance across fish in both hindgut sections sampled (section IV = 39.1% ± 17.49 SD and section V = 54.9% ± 16.48 SD), potentially resulting in variable metabolic significance across fish replicates (Fig. 4B). The higher average relative abundance of *Alistipes* in section V (albeit not a statistically significant difference, Kruskal–Wallis *P* value = 0.25) (Table S12) may also reflect different stages of glycan utilization along the fish gut. These results are supported by our previous 16S rRNA gene analysis of 60 *K. sydneyanus* fish showing that the genus *Alistipes* was significantly more abundant in section V than in sections III and IV [13].

### Brown algae and starch/glycogen are degraded by the wider microbial community

**Figure 5:**
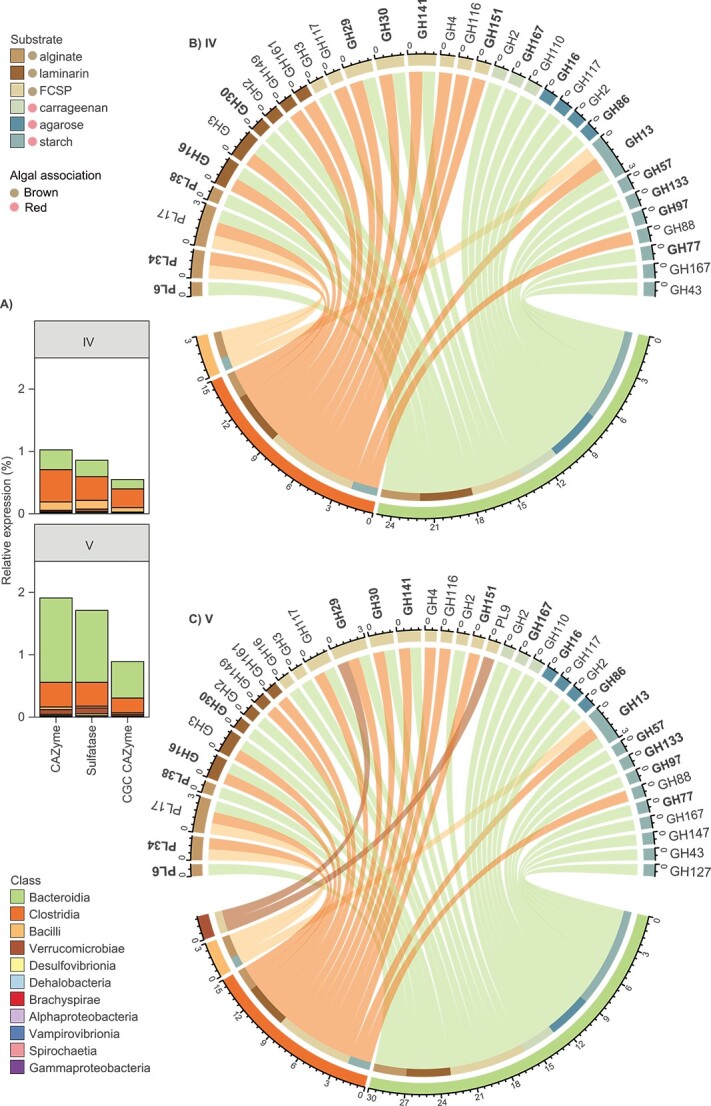
Expression of carbohydrate-associated elements (2017 and 2020 representative community recovered from sections IV and V). (A) Stacked bar plots showing the relative gene expression per section for CAZymes, sulfatases, and CAZymes within CGCs. Bars are coloured according to class**.** (B and C) Chord diagram of highly expressed degradative CGCs (containing at least two CAZymes annotated as GH, PL, or CE) in sections IV (B) and V (C) considering the total CGC expression per section (details in Table S13). CAZy families harbouring HMW activities are shown in bold. CGCs with substrate predictions that comprise the upper quarter of total CGC expression in any sample are shown. Sectors in the upper arc represent the relative expression of CAZy family genes within predicted CGCs (coloured by substrate). Links are coloured by class and associate the relative expression of CAZy family genes to lower arc sectors: bacterial class (middle track) and substrate (inner track).

**Figure 6:**
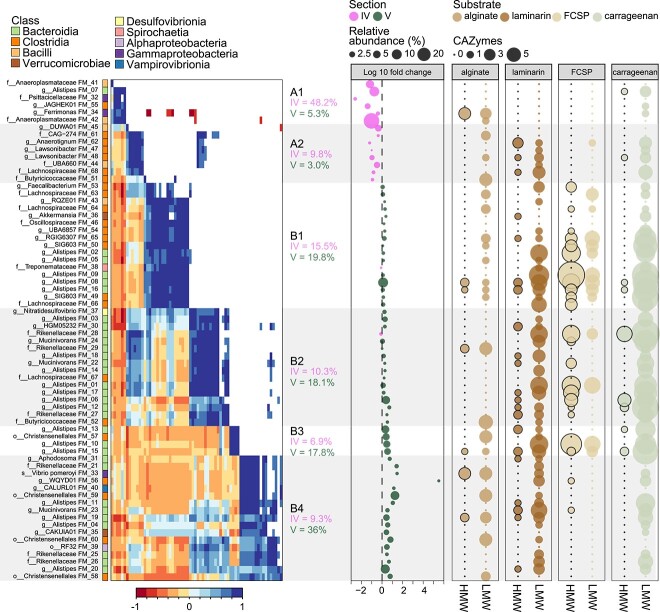
MAG co-abundances and CAZyme enrichment across co-abundant taxon groups. Leftmost plot: Pearson correlation heatmap of MAG relative abundances indicating positive (blue shading) and negative (red shading) correlations (lower triangle). White shading in the upper triangle denotes insignificant *P* values (>0.05). Leftmost labels delineate co-abundant groups and display their group summed relative abundance in sections IV and V. Labels immediately left of the heatmap give MAG IDs and their class affiliation (outer coloured tiles). Middle plot: Log10 fold changes in the relative abundance of each MAG between gut sections. Bubble sizes and colours represent relative abundance and the section the MAG was most abundant. The four right-hand side panels display the CAZyme enrichment in each MAG associated with the utilization of HMW and LMW isoforms of the target polysaccharides.

**Figure 7:**
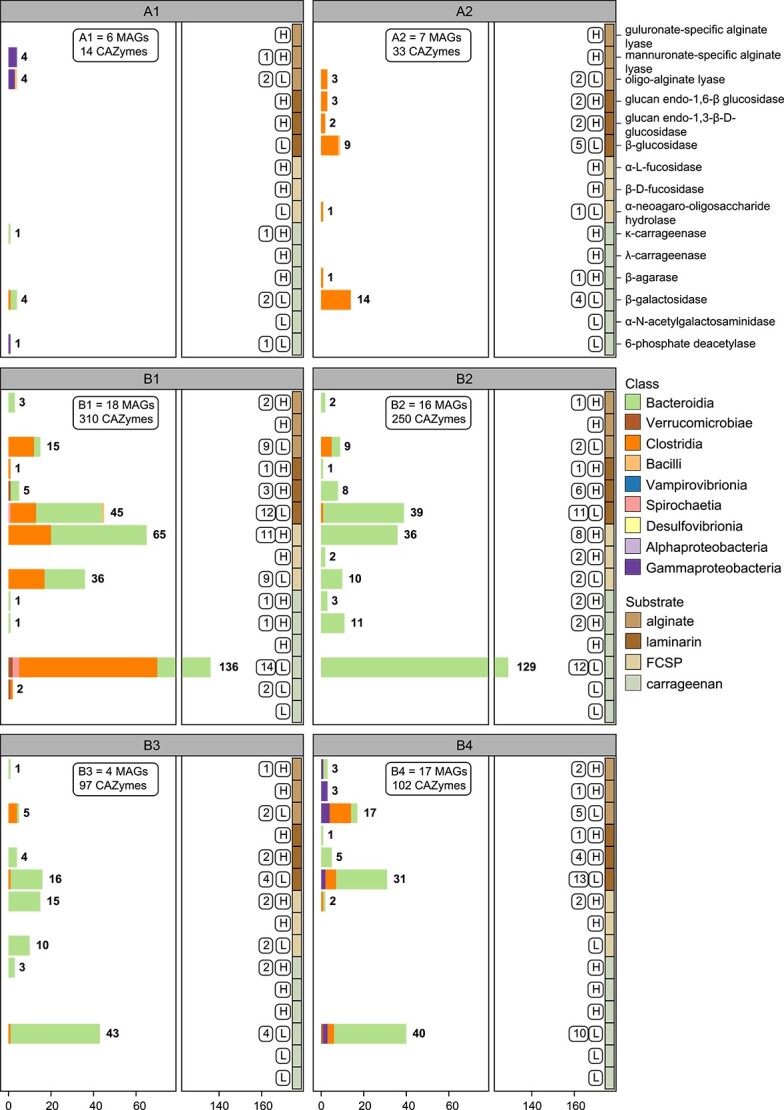
Stacked bar plots showing the pooled count of genes in each co-abundant taxa group per enzyme encoded. Bars show gene counts per taxon class, and numbers directly besides bars give the gene counts. A break was inserted in the *x*-axis to accommodate the large number of galactosidases encoded by *Bacteroidia* MAGs in groups B1 and B2. Coloured tiles on the right represent the substrate each enzyme is predicted to act on. Boxes besides the coloured tiles indicate the number of MAGs encoding specific enzymes, and their capacity for HMW (H) or LMW (L) substrates. Details are given in Table S14.

Gene transcription analysis showed that the breakdown of polysaccharides from brown algae (i.e., alginate, laminarin, FCSP) was primarily undertaken by *Bacteroidia* and *Clostridia* (Fig. 5A) and that members of these classes performed the initiation of alginate (PL6, PL34, and PL38), laminarin (GH16 and GH30), and FCSP (GH29, GH141, and GH151) breakdown in sections IV and V (Fig. 5B and C). As indicated above, other taxa found in the fish hindguts also have the capacity to degrade brown algal polysaccharides. Accordingly, *Bacilli* and *Verrucomicrobiae* were found to express genes for either alginate or FCSP degradation (Fig. 5). Of brown algal substrates, transcriptional data suggest that *Bacilli* primarily degraded alginate in sections IV and V (Fig. 5B and C) [85], despite harbouring other genes associated with brown algae polysaccharide degradation (i.e., of laminarin) (Fig. 2), and other marine-associated *Bacilli* are known to utilize these substrates [85]. The overall low relative abundance of *Verrucomicrobiae* in sections IV and V [13] (0.6 ± 0.9% and 1.2 ± 1.5% on average, Fig. S1A), and relatively low CAZyme-related transcriptional activity (Fig. 5A), suggest this group was not a major contributor to carbohydrate degradation in the gut of *K. sydneyanus* (Fig. 5A). Transcriptional data indicated that *Verrucomicrobiae* primarily acted on FCSP in section V (Fig. 5C), in line with the reported role of members of this lineage in marine environments [73]. Results therefore indicate a niche specialization of these taxonomic groups (*Bacilli* and *Verrucomicrobiae*). Although *Gammaproteobacteria* MAGs (*Vibrio* and *Ferrimonas*) also carried genes for alginate lyases (Figs 2, 4, 6, and 7), we observed lowest expression of associated CAZyme genes in sections IV or V (CAZyme expression <0.001% of total section transcripts, Table S13). Even though *Vibrio* species are well known to degrade alginate and laminarin [86–89], many *Gammaproteobacteria* are generalists able to thrive on diverse substrates [90], facilitating niche differentiation [91]. Many are also considered opportunists [92]. This could explain the *Gammaproteobacteria*-dominated enrichments observed in bioreactor incubations of *K. hawaiiensis* and *K. cinerascens* gut contents and algal polysaccharides [16], and the reduced abundance of *Bacteroidia*, which are prevalent in hindgut communities of *Kyphosus* species [13, 15, 93], as in *K. sydneyanus* (Figs 1A and S1A).

 Transcriptional data indicate that galactose-rich polysaccharides from red algae (such as agarose and carrageenan) were primarily broken down by *Bacteroidia* in sections IV and V, alongside polysaccharides from brown algae (Fig. 5). The affinity of *Bacteroidia* for red algal polysaccharides is consistent with the wide range of sulfatase genes this class possesses, and relatively high expression by *Bacteroidia* of sulfatases associated with FCSP breakdown (Figs 2 and 5), particularly in the biomass-rich hindgut chamber (section V). However, the expression of α-glucosidase genes associated with GH57 (HMW ⍺-amylase) by *Bacteroidia* and GH13 (HMW pullulanase) by *Bacteroidia*, *Clostridia*, and *Bacilli* in sections IV and V (Fig. 5) suggests the utilization of starch from red algae (present in low amounts in the fish diet) or microbial glycogen stores by all these taxa. Glycogen and starch share a similar structure, and glycogen accumulation is considered a common metabolic strategy among gut commensals to survive in a highly competitive, nutrient-limited, or nutrient-fluctuating environment [94]. Taken together, our results indicate competition for brown algae polysaccharides, and utilization of starch or glycogen stores, across the community, whereas only *Bacteroidia* utilized polysaccharides from red algae.

### Evidence for functional redundancy and niche partitioning among co-abundant taxa

The utilization of glycans is a significant driver of interactions yielding positive and negative synergies within microbial gut communities [95–98]. Previous studies conducted by our group explored the prospect of interactions between *Rikenella* and *Alistipes* cooperating or competing for resources, as a result of their phylogenetic relatedness and co-occurrence [13]. Moreover, the wide range of nonutilized glycans by the *K. sydneyanus* host [7] and long residence time of digesta [4] support a landscape for complex interspecies interactions through resource coworking or competition [98]. We therefore examined pairwise correlations of MAG relative abundances to elucidate the potential for positive and negative relationships based on taxa co-abundance (Fig. 6). A clear delineation was observed among co-abundant MAGs when considering changes in their relative abundance from gut section IV to V. Despite a clear trade-off between *Clostridia* and *Bacteroidia* relative abundances, and transcriptional activity, between these sections (Figs 4B and S2), all six co-abundant groups of taxa (A1 to B4) displayed a diverse assortment of taxonomic affiliations, highlighting the potential for interspecies collaborations (or competition). In particular, the presence of CGC-rich *Alistipes* MAGs, across multiple co-abundant groups (Fig. 6), provides further evidence that this genus is important for carbohydrate metabolism within the *K. sydneyanus* gut.

 We observed the distribution of CAZyme-encoding genes for β-glucosidases (laminarin LMW), galactosidases (FCSP/carrageenan LMW), and oligo-alginate lyases (alginate LMW) across co-abundant groups and numerous taxa (Fig. 6). Our results indicate a high level of functional redundancy in the gut communities based on the taxonomically widespread and relatively abundant exo-acting CAZyme capacity. Such redundancy likely confers the microbial community with flexibility in handling a diverse diet [98, 99] and may be facilitated by niche partitioning (e.g., based on variations in enzyme efficiencies or substrate prioritization depending on the nutrient landscape) [95, 98].

The group-wise changes in relative abundance from gut section IV to V presumably reflect a sequential breakdown of glycans along gut sections by taxa in the A groups (exclusively more abundant in section IV) and B groups (members abundant equally in sections IV and V or enriched in V) (Figs 6 and S3A). The emergence of B groups along the gut implies the start and progression of intensive glycan metabolism in gut section IV, which is supported by their large and diverse combined CAZyme capacity (Fig. S3). This coincides with the significantly higher levels of total SCFA in *K. sydneyanus* gut sections IV and V compared to section III (previously reported total SCFA concentrations were 19.35 ± 4.3 S.E. in section III, 49.13 ± 2.62 S.E. in section IV, and 58.07 ± 4.62 S.E. in section V) [6]. The A groups, enriched in section IV only, encoded a limited glycan degradative capacity compared to B (Fig. 7). This observation, combined with the sharp decline in relative abundance of the two A groups from section IV to V, indicates that they are outcompeted in section IV (Figs 7 and S3B). Nonetheless, the high relative abundance in section IV of group A1 taxa, *Bacilli* (FM_42), which encodes an oligo alginate lyase, and *Bacteroidia* (FM_07), which has one κ-carrageenase and four β-galactosidases genes, suggest that alginate and carrageenan metabolism could commence in section III before increasing in later sections. Previous work demonstrated low enzymatic activity against alginate and κ-carrageenan (≤1 μg reducing sugar ml^−1^ min^−1^) between sections II and III [7]. Group A2 MAGs displayed a slightly broader carbohydrate capacity attributed primarily to *Clostridia*, potentially enabling the utilization of alginate oligosaccharides, laminarin, and carrageenan (Figs 6 and 7). MAGs in this group also displayed moderately positive correlations in relative abundance with subsets of MAGs from groups B2 and B4, which share a common capacity for laminarin and carrageenan degradation (via α- and β-desulfated galactans) (Fig. 6).

 The four B groups encode a diversified carbohydrate degradation capacity, with a large enrichment in encoded enzymes, such as β-galactosidases, α-fucosidases, and β-glucosidases (Figs 6 and 7). Their rich CAZyme repertoires explain their maintained or increased relative abundance between gut sections IV and V. Despite the broadly analogous carbohydrate degrading potential among B groups, B1 demonstrated the widespread presence of CAZymes within *Clostridia* MAGs that are associated with the breakdown of alginate, laminarin, and FCSP. In contrast, group B2 comprised mainly *Bacteroidia*, including three MAGs with the highest number of carrageenase genes (for HMW carrageenan) across all co-abundant groups (Figs 6 and 7). This may have provided an advantage to B2 members in sourcing galactose from carrageenan in addition to FCSP. In contrast, only two B2 MAGs carried alginate lyases, suggesting little preference of this group for alginate (Fig. 6). The large proportion of *Bacteroidota*, including seven *Alistipes* MAGs, within B2 implies a metabolism driven by CGCs given the substantial enrichment of CGCs in the genus *Alistipes* (Fig. 3). Members of group B2 may thus have benefited from the adaptive regulation afforded by CGCs to rapidly changing nutrient conditions [52].

Groups B3 and B4 exhibited a tendency for greater relative abundance in hindgut section V. Group B3 consisted of only four MAGs comprising *Alistipes* (*n* = 3) and *Christensenellales* (*n* = 1). These MAGs encoded a relatively large number of CAZyme genes (*n* = 97), despite there being far fewer MAGs in this group (CAZyme-encoding genes were present at a frequency of 24 per genome in group B3 and 6 to 17 per genome in the other B groups) (Fig. 7). However, group B3 lacked the capacity to produce some key enzymes, including glucan endo-1,6-β-glucosidases and mannuronate-specific alginate lyases that are involved in the initial depolymerization of laminarin and alginate, respectively [9]. As a result, bacteria in this group may not efficiently source nutrients from these glycans on their own and potentially require an initial hydrolyzation of laminarin and alginate to thrive. This may explain their increase in abundance in section V, where depolymerized forms of laminarin and alginate are present as a result of previous laminarinase and/or alginate lyase activity [7], presumedly from groups A1 or A2, or perhaps host-derived in the case of laminarin [7]. In addition, Group B3 could be supported by substrates derived from other B groups encoding mannuronate-specific alginate lyase (B4) or glucan endo-1,6-β-glucosidases (B1, B2, and B4) (Fig. 7). Overall, the carbohydrate-degrading capacity in B3 and B4 suggested distributed capacity for LMW substrates across these groups. Whereas B4 exhibited greater capacity towards HMW substrates, only B3 possessed carrageenases, and B4 harboured substantially fewer fucosidases, indicating a deficiency in utilizing galactose on its own (Figs 6 and 7).

Our results highlight the widespread presence and abundance of genes-encoding enzymes targeting LMW carbohydrates across taxa in gut sections IV and V (610 genes across 60 MAGs), enabling utilization of the breakdown products from a smaller group of taxa that are capable of breaking down HMW substrates (184 genes across 38 MAGs) (Table S11). Despite the redundancy observed across the gut community, nuances in CAZyme encoding may confer competitive advantages to each taxon, depending on the nutrient landscape, that regulate the relative abundance of overlapping groups, or their presence–absence, along the intestinal tract of *K. sydneyanus* (Fig. S3A and B). These overlapping groups are likely able to coexist by utilizing alternative nutrient sources or prioritizing substrates to avoid direct competition [52, 95, 98].

These results provide theoretical evidence for interspecies collaborations based on the diverse array of taxa co-occurring with complementary carbohydrate degradation capacities, whereby multiple *Bacteroidia* and *Clostridia* are indicated to initiate polysaccharide breakdown. These observations are in line with the established roles performed by these groups degrading complex polysaccharides in marine ecosystems [37, 57], *Kyphosus* fish [15, 18, 20], and other herbivorous fish [69, 70]. Further, results concur with existing models of microbial interactions between ‘extracellular degraders’ and ‘scavengers’, where the scavengers rely on the extracellular degraders for LMW products [65, 71], and agree with glycan-based niche partitioning literature on the effect of substrate availability [95, 100], complexity [98], and prioritization/preference [91, 95] on microbial composition and interactions. The microbiome capacity of marine herbivorous fishes remains largely unexplored, and few studies have utilized genomics to investigate organisms in the fish gut [14–18, 69, 70]. These are crucial for elucidating the metabolic potential and gene expression profiles *in situ*, providing a foundation for delineating and interpreting *ex situ* studies. Cultivating host-adapted gut communities of wild fish *ex situ* encompasses significant challenges in enriching for and studying dominant species. This difficulty has often led studies to highlight the capabilities of gut taxa derived from *Kyphosus* species, which may not fully replicate the natural gut environment (e.g., integrating microbe/host/environment interactions), as discussed in this study [16, 18, 20]. However, such studies are worth pursuing further to provide evidence of gut microbiome function, alongside corroboration of findings based on *in situ* studies such as this one.

## Conclusions

Our results demonstrate a large capacity for microbial carbohydrate degradation along the *K. sydneyanus* gut associated with the fish diet of brown and red macroalgae. *Bacteroidia* (including many CGC-rich *Alistipes*) and *Clostridia* MAGs contained the highest CAZyme-encoding gene densities in the community, were relatively the most abundant taxa groups, and were responsible for most CAZyme-associated gene transcription. These bacterial classes were predicted to encode most endo-acting CAZymes essential for initiating glycan depolymerization, whereas *Bacilli*, *Verrucomicrobiae*, and *Gammaproteobacteria* had fewer genes for this process. The CAZyme gene repertoires of *Clostridia*, *Bacilli*, *Verrucomicrobiae*, and *Gammaproteobacteria* suggest they primarily consume brown algae, whereas *Bacteroidia* are equipped with a wide variety of genes to degrade both brown and red algae. Accordingly, *Clostridia* and *Bacteroidia* together were responsible for the majority of CAZyme-related gene expression targeting an array of substrates in brown algae (alginate, laminarin, FCSP), and *Bacteroidia* was almost exclusively responsible for gene expression associated with the degradation of red algae (carrageenan, agarose, starch). Analysis of MAG co-abundance dynamics between gut sections IV and V revealed multiple bacterial cohorts capable of degrading dietary carbohydrates. The higher prevalence of *Clostridia* and *Bacilli* in groups more abundant in the proximal gut section suggests they play a role in initiating polysaccharide utilization prior to *Bacteroidia* and is consistent with their relatively high transcriptional activity in section IV. The array of galactosidases and β-glucosidases encoded across members of each cohort indicates their ability to compete for resources and substantial functional redundancy in CAZyme-encoding in the fish gut. The range of CAZymes encoded per cohort also indicates high versatility in glycan utilization and thus the capacity for niche differentiation. Accordingly, results indicate the potential for interspecies collaborations based on the diverse assortment of taxa that co-occurred with complementary carbohydrate degradation potentials, although further investigations are required to confirm the exact substrates of predicted CAZyme-encoding genes and metabolic handoffs among gut bacteria. Multiple *Clostridia* and *Bacteroidia* taxa can thus contribute to the degradation of *E. radiata*, *C. maschalocarpum*, *G. macrocarpa*, and *C. ustulatus* in the *K. sydneyanus* hindgut and are of importance for host nutrition.

## Supplementary Material

Facimoto_2024_Supplementary_Information_ycae102(1)

Facimoto_2024_Supplementary_Tables_ycae102(1)

## Data Availability

Metagenomic data were deposited with NCBI under BioProject PRJNA1029302 (MAG accession numbers are given in Table S3). R codes used for the analysis of this manuscript are available at https://github.com/facimotoCT/Fish_gut_carbohydrate_capacity/.
